# What makes a house a home? Nest box use by West European hedgehogs (*Erinaceus europaeus*) is influenced by nest box placement, resource provisioning and site-based factors

**DOI:** 10.7717/peerj.13662

**Published:** 2022-07-04

**Authors:** Abigail Gazzard, Philip J. Baker

**Affiliations:** School of Biological Sciences, University of Reading, Reading, Berkshire, United Kingdom

**Keywords:** Conservation, Urban ecology, Hedgehogs, Nest boxes, Garden wildlife, Citizen science, Artificial refuge

## Abstract

Artificial refuges provided by householders and/or conservation practitioners potentially represent one mechanism for mitigating declines in the availability of natural nest sites used for resting, breeding and hibernating in urban areas. The effectiveness of such refuges for different species is, however, not always known. In this study, we conducted a questionnaire survey of UK householders to identify factors associated with the use of ground-level nest boxes for West European hedgehogs (*Erinaceus europaeus*), a species of conservation concern. Overall, the percentage of boxes used at least once varied with season and type of use: summer day nesting (35.5–81.3%), breeding (7.2–28.2%), winter day nesting (20.1–66.5%) and hibernation (21.7–58.6%). The length of time the box had been deployed, the availability of artificial food and front garden to back garden access significantly increased the likelihood that a nest box had been used for all four nesting types, whereas other factors related to placement within the garden (*e.g.*, in a sheltered location, on hardstanding such as paving, distance from the house) and resource provisioning (bedding) affected only some nesting behaviours. The factors most strongly associated with nest box use were the provisioning of food and bedding. These data suggest, therefore, that householders can adopt simple practices to increase the likelihood of their nest box being used. However, one significant limitation evident within these data is that, for welfare reasons, householders do not routinely monitor whether their box has been used. Consequently, future studies need to adopt strategies which enable householders to monitor their boxes continuously. Ultimately, such studies should compare the survival rates and reproductive success of hedgehogs within artificial refuges versus more natural nest sites, and whether these are affected by, for example, the impact of nest box design and placement on predation risk and internal microclimate.

## Introduction

The construction of urban areas is typically associated with the loss, fragmentation and degradation of natural habitats (Fischer & Lindenmayer, 2007; Angel et al., 2011). Such changes frequently result in a reduction of the availability and quality of fundamental resources for wildlife including suitable sites for breeding, resting or hibernating (Berthier et al., 2012; Shanahan et al., 2014; Reynolds et al., 2019). Within urban areas, these sites can be lost because of changes at different spatial scales and for different underlying reasons. For example, areas of woodland and scrub may be cleared for building developments, native vegetation may be replaced with non-native species for aesthetic reasons, and individual trees may be removed where they affect built structures or pose a threat to human safety (Reynolds et al., 2019; Wu, Liang & Li, 2019). The loss or degradation of these sites can have significant impacts on populations if associated directly with reproductive output (Franco, Marques & Sutherland, 2005; Chace & Walsh, 2006; Martin & Martin, 2007; Shanahan et al., 2014), but can also disrupt resting (Auslebrook et al., 2018; Grunst et al., 2021) and hibernation patterns (Grol, Voûte & Verboom, 2011). One approach to help mitigate the loss of natural breeding and resting sites in urbanised areas is the use of artificial refuges.

Artificial refuges have been used as conservation tools in towns and cities (Cowan et al., 2021) to improve habitat connectivity, facilitate species introductions or translocations, and/or monitor species abundance and distributions (Beyer & Goldingay, 2006; Williams et al., 2013; Goldingay, Rohweder & Taylor, 2020). In addition to conservation organisations, individual householders may also provide a range of different types of refuges (*e.g.*, bird nest boxes, bat boxes, insect hotels, toad houses, etc.) in their own gardens on an *ad hoc* basis (Gaston et al., 2005; Davies et al., 2009). Clearly, the physical structure of any refuge must meet the requirements of the focal species (*e.g.*, Latham & Knowles, 2008; Kaneko et al., 2010; Hodges & Seabrook, 2016; Rueegger, 2017; Larson et al., 2018; Shaw et al., 2021), but householders can potentially purchase a wide range of commercial designs that vary in size and construction materials. Similarly, designs posted online by conservation or gardening organisations can be equally varied, and householders may also create their own designs based on the materials that are available to them and their own perceptions of species’ requirements.

Well-designed artificial refuges can help to maintain local populations on a long-term basis (Goldingay et al., 2015) and potentially improve breeding success relative to conspecifics using natural nesting sites (Bolton et al., 2004; Libois et al., 2012; Brazill-Boast, Pryke & Griffith, 2013). The provision of nest boxes for the common dormouse (*Muscardinus avellanarius*), for instance, has been associated with a more than doubling in adult abundance (Juškaitis, 2005), and, for urban birds, has been known to aid the recovery of populations to approximately 50% of original levels within five years of the loss of original sites (Dulisz et al., 2022). However, a use of inappropriate designs, materials and/or positioning could result in negative outcomes for wildlife (Larson et al., 2018). For example, nest boxes with poor insulative properties can result in high temperature variability within the nesting chamber (Larson et al., 2015; Larson et al., 2018) which has been associated with declines in clutch size, nestling growth and fledging success of birds (Larson et al., 2015; Bleu, Agostini & Biard, 2017). For lactating mammals, warmer nest boxes could reduce nest attendance (van der Vinne et al., 2014) or occupancy (Guillemette et al., 2008) during breeding periods, but, for hibernators, could help to limit heat loss, and therefore energy expenditure, during torpor (Nedergaard & Cannon, 1990; Madikiza et al., 2010) and aid passive rewarming when rousing (Hoeh et al., 2018). Alternatively, if hibernacula experience unusually high temperatures, the resulting higher body temperatures could lead to substantial increases in energetic expenditure (Humphries, Thomas & Speakman, 2002) as well as more frequent arousals from torpor (Pretzlass & Dausmann, 2012). Different designs may also influence the risk of predation, conspecific parasitism and other forms of disturbance (Davison & Bollinger, 2000). For example, animals using artificial refuges which are more conspicuous than natural nest sites (Evans et al., 2002), or those which lack anti-predator devices (Bailey & Bonter, 2017), might experience increased rates of predation. Furthermore, given that variability in refuge design may ultimately affect survival and/or reproductive rates, species might then be expected to exhibit preferences for those design elements that positively affect these outcomes such as material (Rueegger, Goldingay & Brookes, 2013), entrance type (Goldingay et al., 2015) or orientation (Ardia, Pérez & Clotfelter, 2006). Ultimately, knowledge of these factors would enable conservation practitioners to optimise refuge design.

In addition to their physical design, the use of artificial refuges can be affected by factors relating to positioning (Madikiza et al., 2010) and local- and landscape-level features (Nakamura-Kojo et al., 2014; Le Roux et al., 2016), such as the quality of nearby foraging habitat (Catry et al., 2013) and availability of natural nest sites (Madikiza et al., 2010). Even where good quality habitats are available, however, animals may display a preference for poor quality nest sites that inadvertently induces maladaptive breeding and fitness responses (Battin, 2004; Hale & Swearer, 2016). This concept is termed an ‘ecological trap’ and occurs when there is a mismatch between external cues of nest site selection (*e.g.*, food availability and suitable nest box design) and the actual quality of the site. For example, great tits (*Parus major*) in urban areas preferentially select nest boxes with larger cavities, yet these are associated with lower fledging success (Demeyrier et al., 2016). Ultimately, the provision of artificial refuges may facilitate breeding within sub-optimal habitats (Mänd et al., 2005), and such ecological traps might drive reductions in species abundance (Battin, 2004; Hale & Swearer, 2016).

An absence of knowledge of what constitutes effective design, appropriate placement and whether artificial refuges are used successfully can limit conservation efforts (Cowan et al., 2020). Artificial refuges have been most widely studied (Brady, Risch & Dobson, 2000; Cowan et al., 2021) and applied (Gryz, Jaworski & Krauze-Gryz, 2021) for birds both during and outside of breeding seasons (*e.g.*, Mainwaring, 2011); data relating to ground-dwelling terrestrial small mammals are comparatively limited (Cowan et al., 2021) despite declines within urban areas (*e.g.*, Baker et al., 2003; Gortat et al., 2014; Łopucki & Kitowski, 2017). For example, in the UK, householders are commonly urged by wildlife organisations to install nest boxes in their gardens for the West European hedgehog (*Erinaceus europaeus,* hereafter ‘hedgehog’) (BBC, 2014; British Hedgehog Preservation Society, 2021; The Wildlife Trusts, 2021), a small (<1.5 kg), solitary, nocturnal hibernator of conservation concern (Mathews et al., 2018; Mathews & Harrower, 2020).

Hedgehog numbers have declined substantially in rural areas in the UK over the last few decades (Roos, Johnston & Noble, 2012), which is likely attributable to agricultural intensification (Hof & Bright, 2010; Yarnell & Pettett, 2020), vehicle collisions (Wright et al., 2020), direct predation by or intraguild competition with the European badger (*Meles meles*) (Young et al., 2006; Trewby et al., 2014; Williams et al., 2018a) and habitat loss and fragmentation (Rondinini & Doncaster, 2002; Moorhouse et al., 2014): the latter is most commonly discussed in the context of the loss of hedgerows, a habitat feature that is thought to be particularly important for hedgehogs for nesting, foraging or providing cover from predators (Hof, Snellenberg & Bright, 2012; Pettett et al., 2017). Conversely, however, hedgehogs seem to be attracted to (Doncaster, Rondinini & Johnson, 2001; Pettett et al., 2017) and abundant within (Hubert et al., 2011; Van de Poel, Dekker & Van Langevelde, 2015; Schaus et al., 2020; Schaus Calderón, 2021) areas of human habitation where residential gardens are a widely-used and favoured habitat (Baker & Harris, 2007; Hof & Bright, 2009; Dowding et al., 2010; Williams, Stafford & Goodenough, 2015; Pettett et al., 2017; Williams et al., 2018b; Rasmussen et al., 2019; Gazzard & Baker, 2020; Gazzard, Yarnell & Baker, 2022). Residential gardens (private, typically enclosed, areas adjoining dwellings that may contain lawn(s), ornamental plantings, vegetable plots, ponds, paved areas, decking, sheds and/or other outbuildings) in Britain average 188 m^2^ in size (Office for National Statistics, 2020) but collectively can form up to 47% of total green space in some cities (Loram et al., 2007). In this habitat, householders commonly leave out food for hedgehogs in the form of soft (*e.g.*, canned pet or hedgehog foods) or solid (*e.g.*, pet kibble) products, though the natural diet of hedgehogs is primarily insectivorous (Haigh, Butler & O’Riordan, 2012a; Haigh, O’Riordan & Butler, 2012b). Given that householders have a high affinity for hedgehogs (Morris, 2018), the implementation of hedgehog-friendly activities within gardens could have a significant positive impact on this species.

**Figure 1:**
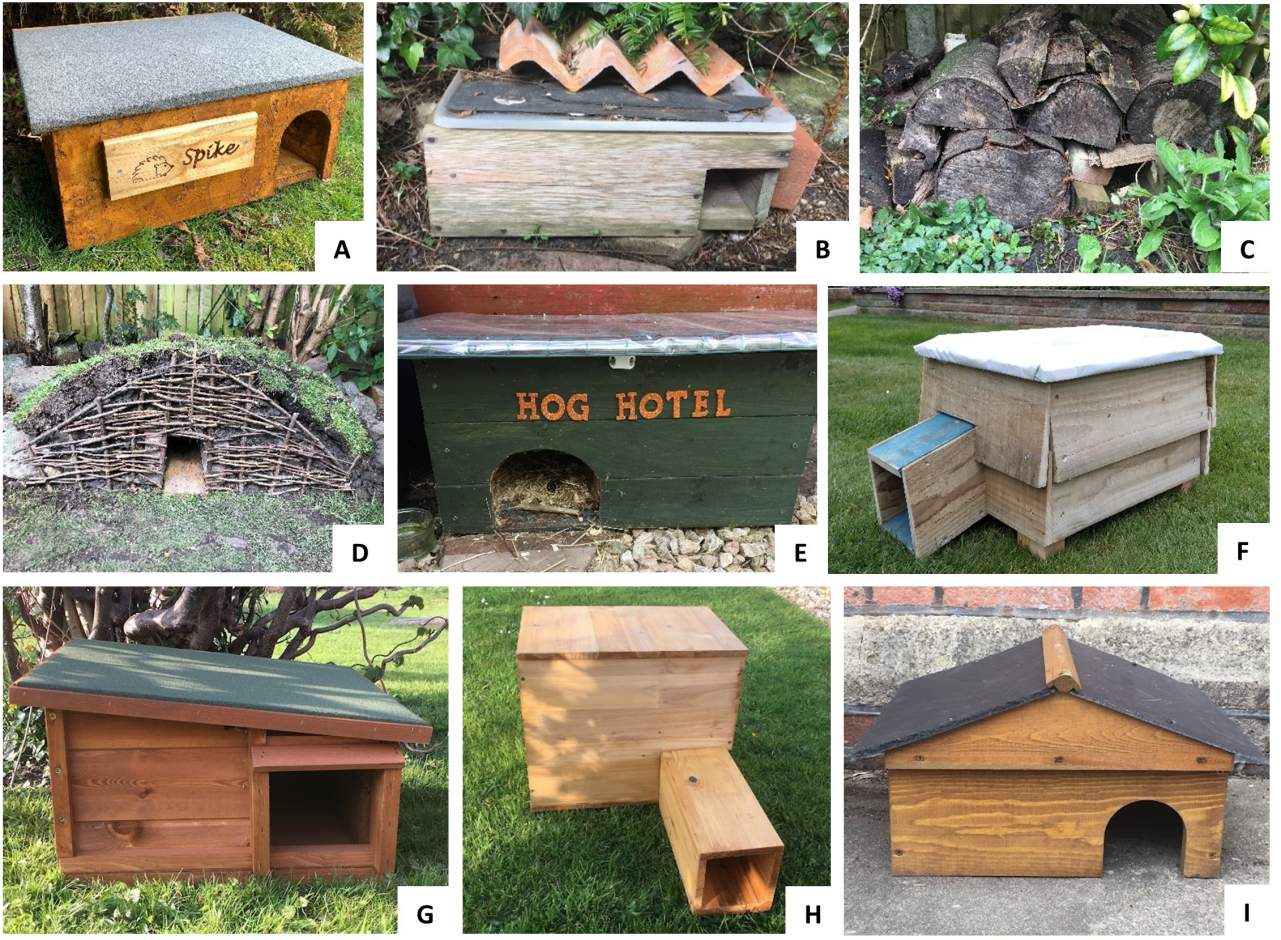
Examples of homemade (A–F) and commercially available (G–I) artificial refuges for hedgehogs. Hedgehog nest boxes vary in size and design, but average nest box dimensions reported in this study (*N* = 4, 509) were 629 × 726 × 423 mm (width ×depth ×height) for homemade nest boxes, and 499 × 478 × 311 mm for manufactured nest boxes. Image credits: (A) G. Northcott; (B) C. Gazzard; (C) S. Wilkinson; (D) L. Pearse; (E) V. Yates; (F) R. Brenton. Commercially available nest box images: A. Gazzard; produced by (G) Home & Roost, (H) Coopers of Stortford and (I) Tom Chambers.

Hedgehog nest boxes (also known as hedgehog houses; Fig. 1) are thought to have grown in popularity over recent years and are now widely commercially available (see Stone, 2020), with numerous guidelines on how to construct homemade versions also available online (*e.g.*, British Hedgehog Preservation Society, 2021; Hedgehog Street, 2021; The Wildlife Trusts, 2021). These are simple boxes or box-like structures within which hedgehogs construct nests out of vegetative material found in the environment or supplied by householders. To date, no studies have been conducted to quantify the frequency with which hedgehogs use nest boxes, whether certain design features or positioning influence their use, and whether influencing factors change between seasons. Such information is fundamental for advising householders and conservation practitioners on how to most effectively provide refuges.

Throughout the annual cycle, hedgehogs construct nests for four different purposes: daytime nesting outside the hibernation period (hereafter ‘summer day nesting’ for brevity), breeding, daytime nesting during the hibernation period (hereafter ‘winter day nesting’) and winter hibernation. In the UK, hedgehogs hibernate between November-April, but typically rouse several times (Reeve, 1994; Morris, 2018). Summer day nesting is therefore defined as day nesting at any time during March-October inclusive. Breeding nests may also be formed at any point during this period: the majority of litters in the UK are produced early in the annual cycle (May-June: Deanesly, 1934; Jackson, 2006; Haigh, 2011), although ‘late litters’ are not uncommon in August-October (Dowler Burroughes, Dowler & Burroughes, 2021). Breeding nests tend to be occupied by the mother and usually 3–6 young (Morris, 1977; Kristiansson, 1981; Walhvod, 1984), with the litter becoming independent by approximately six weeks of age (Morris, 2018).

Rural hedgehogs appear to favour nesting in hedgerows and woodlands (Haigh, O’Riordan & Butler, 2012b; Bearman-Brown et al., 2020); knowledge of nest site selection in urban habitats is lacking, but hedgehogs have been known to nest in gardens (British Hedgehog Preservation Society, 2022). Nests are constructed at ground level under shelter, for example, under hedging, scrub or log piles, though are sometimes also formed in rabbit (*Oryctolagus cuniculus*) burrows or cavities beneath garden sheds or decking (Jackson & Green, 2000; Morris, 2018). Day nests are loosely formed (Rautio et al., 2014) compared to the compact hibernacula or larger breeding nests (Reeve, 1994; Morris, 2018). In all cases, a central nesting core is created under a dome of bedding material that typically contains dry broadleaves, grass and other foliage (Reeve & Morris, 1985; Rautio et al., 2014; Pettett et al., 2017). Nest-sharing by adults is very rare (Reeve, 1994), but hedgehogs will readily make use of multiple day and hibernation nests (Haigh, O’Riordan & Butler, 2012b; Rautio et al., 2014; Morris, 2018; Yarnell et al., 2019; Bearman-Brown et al., 2020), including those that have been used by other individuals (Riber, 2006; Haigh, O’Riordan & Butler, 2012b; Rautio et al., 2014). Distances between successive nest sites have been recorded to range from 2–323 m (Jensen, 2004; Yarnell et al., 2019).

Nesting at ground level exposes hedgehogs to numerous risks: they can be disturbed by humans, companion animals or livestock (Bearman-Brown et al., 2020); are potentially accessible to predators including badgers, foxes (*Vulpes vulpes*) and domestic dogs (*Canis lupus familiaris*); are vulnerable to damage or destruction caused by activities associated with land management, garden maintenance (*e.g.*, mowing or strimming/weed whacking) and construction works; and flooding (Morris, 2018). Disturbances including noise, light or the accidental uncovering of a nest can prompt hedgehogs to relocate nests more frequently than usual, thereby increasing energetic expenditure (Rast, Barthel & Berger, 2019) as well as the risk of mortality from companion animals and road traffic, but can also potentially cause the abandonment or killing of young (Morris, 2018). Furthermore, hedgehogs are likely to be sensitive to changes in the internal microclimate of nests: high levels of humidity have been linked to the advanced decay of broad-leaved nesting material (Morris, 1972), ectoparasite presence (Heeb, Kölliker & Richner, 2000) and the efficiency of animal evaporative heat loss mechanisms (McComb et al., 2021). During hibernation, low temperatures inside nests can accelerate heat loss of individuals in torpor and cause fat stores to rapidly deplete (Soivio, Tähti & Kristoffersson, 1968; Jensen, 2004; Morris, 2018). Last, if different individuals use or reuse the same box in quick succession, this could potentially increase the risk of parasite transmission (Tomás et al., 2007).

Given the lack of quantified information on many aspects relating to the deployment of hedgehog nest boxes by householders, and their use by hedgehogs, we used a questionnaire survey of householders in the UK to identify the factors associated with the use of hedgehog boxes for summer day nesting, breeding, winter day nesting and hibernation. Based on these results, and the associated limitations of these data, we make suggestions for future studies to collect data that would form the basis for recommendations made to householders about how to optimise the deployment of nest boxes (and associated materials such as bedding), as well as identifying the role that artificial refuges may have in the future conservation of this species.

## Materials & Methods

Data were collected using an online questionnaire survey (15/08/2017-31/10/2017 inclusive) of UK householders who had installed at least one hedgehog nest box in their garden. The questionnaire was promoted as ‘The Hedgehog Housing Census’ by the Hedgehog Street campaign (run jointly by the People’s Trust for Endangered Species and British Hedgehog Preservation Society) and advertised through social media, local radio interviews and newspaper articles. As some of the information requested may have led respondents to be tempted to open their box(es) to see if hedgehogs were using them, a statement was included at the start of the questionnaire that explicitly instructed respondents not to do so as this may have adverse effects on the welfare of nesting animals. Additionally, since this survey was focussed on the use of nest boxes as refuges for sleeping, breeding and/or hibernating, a second statement outlined that respondents were not to complete the survey if they had installed hedgehog nest boxes solely for the purpose of feeding hedgehogs: many householders in the UK use covered feeding stations to protect hedgehogs from other species (*e.g.*, cats (*Felis catus*), foxes and badgers) whilst simultaneously protecting the food from inclement weather and from competition with these other species (see Finch et al., 2020). Householders with >1 nest box were asked to complete one survey for each box. Responses that were incomplete, or those that appeared to describe nest boxes being used in locations other than domestic gardens (*e.g.*, in wildlife rescue centres or for rehabilitated individuals at ‘soft release’ sites), were removed prior to analyses.

The questionnaire requested information on a wide range of variables including: the respondent’s geographical location; characteristics of their garden; whether they owned a dog; the size and design features of the box; the length of time that the box had been installed and how it was positioned in the garden; if and where, relative to the location of the nest box, the respondent put food out for hedgehogs; whether they had put any natural or artificial bedding material in the box; whether they fed garden birds; whether they had seen foxes and/or badgers in their garden; and whether the nest box had ever been used by hedgehogs for day nesting in the summer (March-October) or winter (November-February), breeding or hibernating. Nest box use was defined on the basis of direct or indirect observations (*e.g.*, through the use of motion-activated trail cameras or other monitoring devices) of a hedgehog entering or exiting the box. The aerial coverage of urban, arable, woodland and grassland habitat within a 500 m radius around each site (500 m was chosen given existing hedgehog movement data: (Dowding et al., 2010; Schaus Calderón, 2021; Gazzard, Yarnell & Baker, 2022)), and the distance to the nearest patch of each of these habitats, were quantified with PostGIS and using a 2017 Land Cover Map of the UK (UK Centre for Ecology & Hydrology; Morton et al., 2020); the aggregate habitat classes in the Land Cover Map are based on Broad Habitat Classifications used in the government’s 1994 UK Biodiversity Action Plan (Jackson, 2000). Variables are defined in Table 1.

**Table 1:** Variables considered in the analysis of hedgehog nest box use within gardens. Source: Q = the variable was derived from the questionnaire survey; LC = data were quantified using a UK land class dataset (Morton et al., 2020).

Theme	Variable	Description	Variable type	Source
Nest box use	SUMMERDAY BREEDING WINTERDAY HIBERNATION	How long, in months, the respondent believed that the nest box had been used for summer day nesting (March–October), breeding, winter day nesting (November–February) or hibernation	Converted to binary variable for analysis: (0) Box had not been used (1) Box had been used	Q
Time installed	MONTHS INSTALLED	The number of months the nest box had been installed in the respondent’s garden	Continuous	Q
Nest box design	TYPE	Whether the nest box was homemade or purchased	(0) Purchased (1) Homemade	Q
	MATERIAL	The primary material the nest box was constructed with	(0) Timber (1) Plywood/plyboard (2) Brushwood (3) Plastic (4) Other (including wicker, woodcrete and brick)	Q
	HEIGHT	Nest box height (cm)	Continuous	Q
	WIDTH	Nest box width (cm)	Continuous	Q
	DEPTH	Nest box depth (cm) (front to back)	Continuous	Q
	CAPACITY	Maximum capacity (cm^3^) of the nest box (Height * Width * Depth)	Continuous	Q
	BASE	Whether the nest box had an integral base	(0) No base (1) Base	Q
	TUNNEL	Whether the nest box had an external tunnel entrance	(0) No tunnel entrance (1) Tunnel entrance	Q
	PARTITION	Whether the nest box had an internal tunnel or partition	(0) No internal partition (1) Internal partition	Q
	VENT	Whether the nest box had ventilation holes	(0) No vent (1) Vent	Q
	LINING	Whether the nest box had a waterproof lining	(0) Nest box is unlined (1) Nest box is lined	Q
Nest box positioning	FRONT OR BACK	Whether the nest box was positioned in a front or back garden	(0) Front garden (1) Back garden	Q
	HARD STANDING	Whether the nest box was located on hardstanding (*i.e.*, patio, paved or decked areas)	(0) Not on hardstanding (1) On hardstanding	Q
	SHELTERED	Whether the nest box was under shelter (*e.g.,* shrubbery)	(0) Not in sheltered location (1) In a sheltered location	Q
	DISTANCE BUILDING	Whether the nest box was located <5 m from a building	(0) ≥5 m from a building (1) <5 m from a building	Q
	FACING	Whether the nest box entrance was facing into the open or elsewhere	(0) Facing a wall/fence (1) Parallel to a wall/fence (2) Facing shrubs/planting (3) Facing the open (4) Other	Q
	ORIENTATION	The direction that the nest box entrance was facing	(0) North (1) East (2) South (3) West	Q
	RAISED	Whether the nest box was raised off the ground or not	(0) Not raised (1) Raised	Q
Garden characteristics	CONNECTED	The number of neighbouring back gardens which were accessible to hedgehogs from the respondent’s back garden	Continuous	Q
	FRONT BACK ACCESS	Whether a hedgehog could access the respondent’s back garden from their front garden	(0) No front-to-back access (1) Front-to-back access	Q
	OTHER NESTS	Whether the respondent had directly observed, or found evidence of, hedgehogs nesting outside of a nest box elsewhere in their back garden	(0) No alternative nesting sites observed (1) Alternative nesting sites observed	Q
	POND	Whether the garden contained a pond	(0) No pond (1) Pond	Q
	GOOD HAB	The proportion of “good” habitat present in the garden, including shrubs, a wild area, woodpile, compost heap, shed or decking with cavity beneath, lawn, vegetable patch and/or flowerbeds	Continuous (proportion)	Q
Resources for hedgehogs	BEDDING	Whether the respondent provided artificial (*e.g.,* newspaper) or natural (*e.g.,* leaves, hay) bedding within the nest box, or both	(0) None provided (1) One type of bedding (2) Both types of bedding	Q
	HEDGEHOG FOOD	The location in which food was provided for hedgehogs in the garden	(0) None provided (1) Scattered in varying locations (2) <0.5 m from nest box (3) 0.5–5 m from nest box (4) 5.1–10 m from nest box (5) >10 m from nest box	Q
Other animals	BIRD FOOD	Whether the respondent supplied food for birds in their garden	(0) None provided (1) Food provided	Q
	BADGERFOX	Whether the respondent ever observed badgers or foxes in their garden (NB badger and fox sightings were merged due to the low number of positive sightings)	(0) Not sighted (1) Sighted	Q
	DOGS	Whether the respondent owned any pet dogs that were allowed access to the garden	(0) No dogs (1) Dogs	Q
Habitats	URBAN500 ARABLE500 WOOD500 GRASS500	The quantity (m^2^) of urban/arable/woodland/grassland habitats within 500 m of the respondent’s location (estimated from the central point of their postcode)	Continuous	LC
	URBANDIST ARABLEDIST WOODDIST GRASSDIST	Distance (m) to the nearest urban/arable/woodland/grassland habitat patches from the respondent’s location (estimated from the central point of their postcode)	Continuous	LC

To assess factors affecting whether nest boxes had been used by hedgehogs, we fitted generalised linear models (GLMs) with binomial distributions and logit link functions. Many participants were not aware of whether their nest box had been used for every category of nesting but could indicate whether the nest box had been used for at least one type. Therefore, the data were separated into four groups representing nest boxes that were known to have been or not have been used for: (a) summer day nesting, (b) breeding, (c) winter day nesting or (d) hibernation. In addition, as many nest boxes had only been installed recently, the response variable indicated whether the nest box had been used at any point since it had been deployed rather than the extent of use over time. When the binary responses comprised a greater number of “events” (nest box used) than “non-events” (nest box not used), we opted not to balance (subsample the dataset to obtain an even split of events and non-events) the data in favour of treating each of the four GLM analyses in the same way. Furthermore, the imbalances in proportions of boxes used were not extreme, and biases in maximum likelihood estimates are reduced in larger sample sizes (see Jiménez-Valverde, Lobo & Hortal, 2009; Salas-Eljatib et al., 2018).

Correlations between explanatory variables were checked prior to analysis, and further examined using generalised variance inflation factors (GVIFs) during the modelling process (Fox & Monette, 1992). For the former, the threshold of ‘high’ correlation was set at a Pearson coefficient value <−0.5 or >0.5, and for the latter, the GVIF threshold was chosen to be <2 (see Zuur, Ieno & Elphick, 2010). Models were constructed by sequentially adding variables and examining Akaike’s Information Criterion (AIC) values (Burnham & Anderson, 1998). In some cases, nonsignificant variables were retained if they improved model fit and/or if it was considered informative to highlight their importance across different nesting types (*e.g.*, BEDDING, BADGERFOX, DOGS). Final model fit was assessed with the Hosmer-Lemeshow test which compares the number of expected events—as deduced from the regression model—to the number of observed events, commonly for 10 divisions of the dataset (Hosmer & Lemesbow, 1980). Additionally, Nagelkerke pseudo-R^2^ values are provided for each final model to give an indication of the amount of variance in the dependent variable that was explained by the independent variables (Nagelkerke, 1991). Analyses were performed in R 4.0.3.

## Results

In total, 4,309 questionnaire responses were available for analysis: 1,717 (39.8%) responses were associated with homemade nest boxes, and 2,592 (60.2%) with commercially purchased nest boxes. However, 1,492 respondents did not monitor the use of their nest box and could not provide information on whether it had been used for any type of nesting. Considering only those respondents who stated that they knew whether their nest box had been used or not, 81.3% (*N* = 1, 868), 28.2% (*N* = 1, 104), 66.5% (*N* = 1, 300) and 58.6% (*N* = 1, 592) reported that hedgehogs had used the box at least once for summer day nesting, breeding, winter day nesting or hibernation, respectively.

Of all nest boxes reported, 46.3% had been installed for <1 year, 42.0% for 1–5 years, 8.2% for 5–10 years and 3.6% for >10 years. Overall, 77.9% of homemade boxes and 79.5% of commercial boxes had been installed only after hedgehogs had been sighted in the garden: this is equivalent to 78.9% of all the boxes in the survey. Respondents also collectively directly observed, or observed evidence of, at least 2,546 other nest sites used by hedgehogs within their gardens, which equates to an average of 0.6 per garden. These comprised nests constructed under garden vegetation (46.0%), woodpiles (14.5%), compost heaps (6.3%), decking (5.7%), sheds (21.5%) and buildings (6.1%).

### Factors affecting nest box use

The number of survey responses that were available for modelling factors affecting the use of nest boxes varied between nesting types: 1,868 for summer day nesting, 1,104 for breeding, 1,300 for winter day nesting, and 1,592 for hibernation. Multicollinearity checks showed that some of the nest box design variables, as well as habitats variables, were correlated. Consequently, the following variables (Table 1) were excluded from the analyses: BASE, TUNNEL, PARTITION, VENT, LINING, ARABLE500, WOOD500, and GRASS500. In addition, URBANDIST was omitted as most participants lived directly within urban areas; <6% of respondents resided outside of land classified as urban.

For all types of nest box use, the length of time it had been installed, positioning within the garden, the provisioning of resources and site-based factors significantly influenced whether it had been used or not (Tables 2 and 3). Nest boxes were significantly more likely to have been used for all four patterns of use the longer they had been installed, if the back garden could be accessed from the front, if the householder put out food for hedgehogs, and if other nest sites were present in the garden (Table 3). Positioning on hardstanding (such as paving, patio or decking), in a sheltered location and the supply of bedding each increased the likelihood that the box was used in three of the four contexts, but these were not consistent. Boxes in close proximity to a building, those that were raised off the ground and were homemade were more likely to have been used in two contexts, but again these patterns were not consistent. Factors that significantly negatively impacted nest box use included whether the entrance to the box faced into the open, the presence of a garden pond, an increase in the extent of potentially valuable habitat (including shrubs, a wild area, woodpile, compost heap, shed or decking with cavity beneath, lawn, vegetable patch and/or flowerbeds) within the garden, and the presence of dogs (Table 3).

**Table 2:** Results of generalised linear models examining factors affecting hedgehog nest box use for (a) summer day nesting, (b) breeding, (c) winter day nesting and (d) hibernation. Reference levels for variables are indicated in parentheses. SE = standard error; OR = odds ratio. Variables that had a significant effect (*p* < 0.05) are highlighted in bold.

**(a)** Summer day nests (*N* = 1,868). Hosmer and Lemeshow Test: *χ*^2^_8_ = 10.226, *p* = 0.250; Nagelkerke *R*^2^ = 0.235.						
*Variable*	*Estimate*	*SE*	*z value*	*p*	*OR*	95% CI
(Intercept)	−1.250	0.524	−2.384	0.017	0.287	0.103–0.810
**MONTHS INSTALLED**	**0.009**	**0.002**	**3.843**	**<0.001**	**1.009**	**1.005–1.015**
TYPE (Purchased)						
Homemade	0.261	0.140	1.860	0.063	1.298	0.988–1.712
**FRONT OR BACK (Front garden)**						
**Back garden**	**0.528**	**0.220**	**2.398**	**0.016**	**1.696**	**1.094–2.599**
**HARD STANDING (Not on hardstanding)**						
**On hardstanding**	**0.430**	**0.190**	**2.266**	**0.023**	**1.538**	**1.068–2.251**
**SHELTERED (Not in sheltered location)**						
**In a sheltered location**	**0.529**	**0.168**	**3.158**	**0.002**	**1.698**	**1.219–2.354**
**DISTANCE BUILDING (≥5 m from a building)**						
**<5 m from a building**	**0.421**	**0.158**	**2.667**	**0.008**	**1.524**	**1.122–2.085**
**FACING (Entrance faces wall/fence)**						
Parallel to wall/fence	0.001	0.302	0.003	0.997	1.001	0.541–1.780
Faces shrubs/plantings	−0.043	0.314	−0.138	0.890	0.957	0.507–1.746
**Faces the open**	**−0.585**	**0.296**	**−1.978**	**0.048**	**0.557**	**0.305–0.976**
Other	−0.267	0.336	−0.794	0.427	0.766	0.390–1.465
**RAISED (Not raised)**						
**Raised**	**0.364**	**0.178**	**2.053**	**0.040**	**1.440**	**1.023–2.054**
**FRONT BACK ACCESS (No front-to-back access)**						
**Front-to-back access**	**0.470**	**0.143**	**3.277**	**0.001**	**1.599**	**1.206–2.116**
**OTHER NESTS (No alternative nesting sites used)**						
**Alternative nesting sites used**	**0.768**	**0.136**	**5.650**	**<0.001**	**2.156**	**1.655–2.821**
**POND (No garden pond)**						
**Pond**	**−0.368**	**0.141**	**−2.602**	**0.009**	**0.692**	**0.525–0.914**
**GOOD HAB**	**−0.012**	**0.004**	**−2.982**	**0.003**	**0.988**	**0.980–0.996**
**BEDDING (None provided)**						
**One type**	**0.914**	**0.182**	**5.008**	**<0.001**	**2.493**	**1.740–3.560**
**Both types**	**1.457**	**0.268**	**5.431**	**<0.001**	**4.293**	**2.560–7.347**
**HEDGEHOG FOOD (None provided)**						
Scattered in varying locations	0.803	0.510	1.574	0.115	2.233	0.865–6.583
**<0.5 m from nest box**	**0.881**	**0.237**	**3.720**	**<0.001**	**2.414**	**1.523–3.857**
**0.5–5 m from nest box**	**1.463**	**0.185**	**7.903**	**<0.001**	**4.317**	**3.004–6.209**
**5.1–10 m from nest box**	**1.133**	**0.313**	**3.621**	**<0.001**	**3.105**	**1.708–5.849**
**>10 m from nest box**	**1.233**	**0.243**	**5.082**	**<0.001**	**3.433**	**2.145–5.563**
BADGERFOX (Not sighted)						
Sighted	−0.159	0.135	−1.177	0.239	0.853	0.655-1.111
DOGS (Absent)						
Present	−0.224	0.152	−1.471	0.141	0.799	0.594–1.081
**(b)** Breeding nests (*N* = 1,104). Hosmer and Lemeshow Test: *χ*^2^_8_ = 8.621, *p* = 0.375; Nagelkerke *R*^2^ = 0.230.						
*Variable*	*Estimate*	*SE*	*z value*	*p*	*OR*	95% CI
(Intercept)	−3.325	0.422	−7.873	<0.001	0.036	0.015–0.080
**MONTHS INSTALLED**	**0.017**	**0.002**	**7.908**	**<0.001**	**1.017**	**1.013–1.021**
TYPE (Purchased)						
Homemade	0.267	0.153	1.746	0.081	1.306	0.967–1.762
**SHELTERED (Not in sheltered location)**						
**In a sheltered location**	**0.570**	**0.228**	**2.498**	**0.013**	**1.768**	**1.144–2.805**
**FRONT BACK ACCESS (No front-to-back access for hedgehogs)**						
**Front-to-back access**	**0.462**	**0.177**	**2.606**	**0.009**	**1.587**	**1.127–2.259**
**OTHER NESTS (No alternative nesting sites used)**						
**Alternative nesting sites used**	**0.863**	**0.152**	**5.692**	**<0.001**	**2.369**	**1.764–3.196**
**POND (No garden pond)**						
**Pond**	**−0.667**	**0.165**	**−4.041**	**<0.001**	**0.513**	**0.370–0.707**
BEDDING (None provided)						
One type	−0.067	0.229	−0.291	0.771	0.936	0.602–1.480
Both types	−0.229	0.286	−0.804	0.422	0.795	0.455–1.395
**HEDGEHOG FOOD (None provided)**						
Scattered in varying locations	0.992	0.552	1.797	0.072	2.696	0.877–7.810
<0.5 m from nest box	−0.054	0.386	−0.140	0.889	0.947	0.440–2.017
**0.5–5 m from nest box**	**0.976**	**0.280**	**3.485**	**<0.001**	**2.653**	**1.563–4.705**
5.1–10 m from nest box	0.719	0.403	1.784	0.074	2.053	0.926–4.531
**>10 m from nest box**	**1.141**	**0.321**	**3.556**	**<0.001**	**3.131**	**1.691–5.978**
BADGERFOX (Not sighted)						
Sighted	−0.096	0.149	−0.645	0.519	0.908	0.678–1.216
DOGS (Absent)						
Present	−0.323	0.182	−1.776	0.076	0.724	0.504–1.029
**(c)** Winter day nests (*N* = 1,300). Hosmer and Lemeshow Test: *χ*^2^_8_ = 10.681, *p* = 0.220; Nagelkerke *R*^2^ = 0.240.						
*Variable*	*Estimate*	*SE*	*z value*	*p*	*OR*	95% CI
(Intercept)	−1.976	0.516	−3.832	<0.001	0.139	0.050–0.381
**MONTHS INSTALLED**	**0.014**	**0.003**	**5.442**	**<0.001**	**1.014**	**1.009–1.019**
**TYPE (Purchased)**						
**Homemade**	**0.483**	**0.137**	**3.515**	**<0.001**	**1.621**	**1.240–2.125**
**HARD STANDING (Not on hardstanding)**						
**On hardstanding**	**0.468**	**0.184**	**2.547**	**0.011**	**1.597**	**1.119–2.302**
**SHELTERED (Not in sheltered location)**						
**In a sheltered location**	**0.495**	**0.175**	**2.821**	**0.005**	**1.640**	**1.163–2.314**
DISTANCE BUILDING (≥5 m from a building)						
<5 m from a building	0.216	0.148	1.463	0.144	1.241	0.930–1.662
**FACING (Entrance faces wall/fence)**						
Parallel to wall/fence	−0.077	0.285	−0.270	0.787	0.926	0.523–1.605
Faces shrubs/plantings	0.031	0.297	0.104	0.917	1.031	0.570–1.831
**Faces the open**	**−0.590**	**0.279**	**−2.112**	**0.035**	**0.555**	**0.317–0.949**
Other	0.188	0.323	0.582	0.561	1.207	0.637–2.266
**FRONT BACK ACCESS (No front-to-back access for hedgehogs)**						
**Front-to-back access**	**0.366**	**0.150**	**2.445**	**0.014**	**1.441**	**1.074–1.932**
**OTHER NESTS (No alternative nesting sites used)**						
**Alternative nesting sites used**	**0.560**	**0.134**	**4.195**	**<0.001**	**1.751**	**1.349–2.278**
POND (No garden pond)						
Pond	−0.232	0.142	−1.626	0.104	0.793	0.600–1.049
GOOD HAB	−0.006	0.004	−1.441	0.150	0.994	0.987–1.002
**BEDDING (None provided)**						
**One type**	**0.868**	**0.204**	**4.261**	**<0.001**	**2.383**	**1.601–3.564**
**Both types**	**1.484**	**0.266**	**5.585**	**<0.001**	**4.412**	**2.636–7.482**
**HEDGEHOG FOOD (None provided)**						
Scattered in varying locations	0.555	0.498	1.116	0.265	1.742	0.665–4.759
**<0.5 m from nest box**	**0.662**	**0.267**	**2.477**	**0.013**	**1.938**	**1.151–3.283**
**0.5–5 m from nest box**	**1.065**	**0.212**	**5.011**	**<0.001**	**2.900**	**1.916–4.412**
**5.1–10 m from nest box**	**0.633**	**0.306**	**2.071**	**0.038**	**1.883**	**1.038–.447**
**>10 m from nest box**	**1.251**	**0.270**	**4.635**	**<0.001**	**3.492**	**2.069–5.966**
BIRD FOOD (None provided)						
Provided	0.201	0.134	1.507	0.132	1.223	0.941–1.589
BADGERFOX (Not sighted)						
Sighted	−0.219	0.133	−1.643	0.100	0.803	0.618–1.043
DOGS (Absent)						
Present	−0.224	0.152	−1.471	0.141	0.799	0.594–1.081
**(d)** Hibernation nests (*N* = 1,592). Hosmer and Lemeshow Test: *χ*^2^_8_ = 14.175, *p* = 0.077; Nagelkerke *R*^2^ = 0.286.						
*Variable*	*Estimate*	*SE*	*z value*	*p*	*OR*	95% CI
(Intercept)	−2.731	0.486	−5.622	<0.001	0.065	0.025–0.168
**MONTHS INSTALLED**	**0.021**	**0.002**	**8.820**	**<0.001**	**1.021**	**1.017–1.026**
**TYPE (Purchased)**						
**Homemade**	**0.251**	**0.121**	**2.080**	**0.038**	**1.285**	**1.015–1.630**
FRONT OR BACK (Front garden)						
Back garden	0.079	0.221	0.357	0.721	1.082	0.700–1.666
**HARD STANDING (Not on hardstanding)**						
**On hardstanding**	**0.373**	**0.161**	**2.316**	**0.021**	**1.452**	**1.061–1.996**
SHELTERED (Not in sheltered location)						
In a sheltered location	0.299	0.161	1.862	0.063	1.348	0.984–1.848
**DISTANCE BUILDING (≥5 m from a building)**						
**<5 m from a building**	**0.341**	**0.137**	**2.500**	**0.012**	**1.407**	**1.078–1.841**
**FACING (Entrance faces wall/fence)**						
Parallel to wall/fence	0.001	0.302	0.003	0.997	1.001	0.541–1.780
Faces shrubs/plantings	−0.043	0.314	−0.138	0.890	0.957	0.507–1.746
**Faces the open**	**−0.585**	**0.296**	**−1.978**	**0.048**	**0.557**	**0.305-0.976**
Other	−0.267	0.336	−0.794	0.427	0.766	0.390–1.465
**ORIENTATION (Entrance faces North)**						
East	0.305	0.172	1.776	0.076	1.357	0.969–1.902
**South**	**0.347**	**0.168**	**2.065**	**0.039**	**1.415**	**1.018–1.968**
West	0.189	0.185	1.020	0.308	1.208	0.840–1.737
**RAISED (Not raised)**						
**Raised**	**0.364**	**0.178**	**2.053**	**0.040**	**1.440**	**1.023–2.054**
CONNECTED	−0.021	0.045	−0.473	0.636	0.979	0.896–1.070
**FRONT BACK ACCESS (No front-to-back access for hedgehogs)**						
**Front-to-back access**	**0.496**	**0.134**	**3.697**	**<0.001**	**1.643**	**1.263–2.139**
**OTHER NESTS (No alternative nesting sites used)**						
**Alternative nesting sites used**	**0.656**	**0.118**	**5.564**	**<0.001**	**1.926**	**1.530–2.428**
POND (No garden pond)						
Pond	−0.130	0.125	−1.040	0.299	0.878	0.686–1.122
GOOD HAB	−0.004	0.003	−1.173	0.241	0.996	0.989–1.003
**BEDDING (None provided)**						
**One type**	**0.720**	**0.183**	**3.940**	**<0.001**	**2.055**	**1.440–2.952**
**Both types**	**1.107**	**0.237**	**4.677**	**<0.001**	**3.024**	**1.909–4.831**
**HEDGEHOG FOOD (None provided)**						
Scattered in varying locations	0.141	0.477	0.295	0.768	1.151	0.449–2.946
<0.5 m from nest box	0.167	0.245	0.684	0.494	1.182	0.732–1.911
**0.5–5 m from nest box**	**0.933**	**0.188**	**4.957**	**<0.001**	**2.542**	**1.763–3.690**
**5.1–10 m from nest box**	**0.962**	**0.290**	**3.319**	**0.001**	**2.617**	**1.489–4.646**
**>10 m from nest box**	**1.088**	**0.237**	**4.597**	**<0.001**	**2.968**	**1.873–4.739**
BIRD FOOD (None provided)						
Provided	−0.102	0.121	−0.843	0.399	0.903	0.712–1.144
BADGERFOX (Not sighted)						
Sighted	−0.133	0.119	−1.112	0.266	0.876	0.693–1.106
**DOGS (Absent)**						
**Present**	**−0.346**	**0.140**	**−2.462**	**0.014**	**0.708**	**0.537–0.932**

**Table 3:** Summary of explanatory variables considered in GLM analyses that had significant positive (**+**) or negative (**-**) influences on the use of nest boxes by hedgehogs for summer day nesting, breeding, winter day nesting and/or hibernation (see Table 2 for detailed breakdown). Single symbol = *p* < 0.05, double symbol = *p* < 0.01, triple symbol = *p* < 0.001.

Variable	Summer	Breeding	Winter	Hibernation
MONTHS INSTALLED	**+++**	**+++**	**+++**	**+++**
TYPE			**+++**	**+**
MATERIAL				
HEIGHT				
WIDTH				
DEPTH				
CAPACITY				
FRONT OR BACK	**+**			
HARD STANDING	**+**		**+**	**+**
Sheltered	**++**	**+**	**++**	
DISTANCE BUILDING	**++**			**+**
FACING	**-**		**-**	**-**
ORIENTATION				**+**
RAISED	**+**			**+**
CONNECTED				
FRONT BACK ACCESS	**++**	**++**	**+**	**+++**
OTHER NESTS	**+++**	**+++**	**+++**	**+++**
POND	**- -**	**- - -**		
GOOD HAB	**- -**			
BEDDING	**+++**		**+++**	**+++**
HEDGEHOG FOOD	**+++**	**+++**	**+++**	**+++**
BIRD FOOD				
BADGERFOX				
DOGS				**-**
URBAN500				
ARABLEDIST				
WOODDIST				
GRASSDIST				

**Notes.**

For variables with >2 categories, the effect refers to the following levels: Facing the open; Orientation to the south; BEDDING provided was natural, artificial or both; HEDGEHOG FOOD provided in any of the possible locations listed in the survey including scattered, <0.5 m, 0.5–5 m, 5.1–10 m and/or >10 m from the nest box.

## Discussion

The percentage of nest boxes reported to have been used at least once varied between nesting types: summer day nesting (81.3%), breeding (28.2%), winter day nesting (66.5%) and hibernation (58.6%). These are, however, maximum figures because of the way in which data were collated for analysis; we were only able to include respondents who had evidence that their box had, or had not, been used for these purposes. If we assume that all additional boxes owned by respondents who did not have such evidence had never been used, then these figures would be markedly reduced: summer day nesting (35.3%), breeding (7.2%), winter day nesting (20.1%) and hibernation (21.7%). This lack of definitive information about patterns of use is, in part, related to the fact that conservation organisations recommend that householders should not look in their box to check if they are being used due to the risks associated with disturbing hedgehogs. Instead, householders are advised to use motion-activated cameras outside the box or other approaches, such as placing a small stick or piece of straw across the entrance, to determine whether an animal (assumed to be a hedgehog) has entered. Although critically important in the context of ensuring the welfare of the animals involved, this does limit the amount of data available for studies such as this one: we were only able to consider whether nest boxes had ever been used during their ‘lifetime’, rather than the frequency of use.

The majority (88.3%) of hedgehog nest boxes had been installed within the five-year period prior to this survey, implying that there has been a marked increase in recent years in the number of householders providing such refuge structures. In most cases (78.9%), these boxes were deployed after the householder knew that hedgehogs were already visiting their garden implying that personal knowledge of the species’ presence is a particularly strong motivational factor influencing whether householders decide to help hedgehogs in this way. Nevertheless, the subsequent use of these boxes will be dependent on the suitability of their design and placement in the householder’s garden. Consequently, it is important that the factors influencing nest box use for day nesting, breeding and/or hibernating are identified so that householders and conservation practitioners can be advised appropriately to maximise their use.

Collectively, our analyses indicated that there were subtle differences in the factors associated with the use of nest boxes for day nesting, breeding and hibernating, but, overall, these tended to be factors relating to nest box placement, resource provisioning and site-based features, rather than those relating to box design. For all nesting types, the length of time the box had been deployed, the availability of artificial food and the presence of access points for hedgehogs into back gardens from the front significantly increased the likelihood that a nest box had been used. However, both deployment time and artificial food could be associated with a form of reporting bias. For example, householders may have been increasingly more likely to have noticed, by chance, that their box had been used simply because the box had been in their garden for longer, and those householders who fed hedgehogs, may have been more likely to monitor hedgehog activity in their garden; the latter may also explain the positive association between nest box use and the identification of other nesting sites in the garden. Alternatively, it is known that a range of mammal species exhibit neophobic responses to novel objects in the environment (Stryjek, Kalinowski & Parsons, 2019), such that hedgehogs may need to become habituated to a nest box before using it (*sensu* Madikiza et al., 2010).

Conversely, artificial food may represent an attractive resource for hedgehogs in the context of selecting the position of nest sites. Food abundance has been known to influence nest box occupancy by arboreal mammals (Nakamura-Kojo et al., 2014) and, for birds, can facilitate greater occupancy of closely spaced refuges when compared to sites where food is less abundant (Hussel, 2012). Supplementary food can simultaneously act to increase energy intake and reduce foraging time, such that animals would have more time for nest building (Smith et al., 2013). However, patterns of hibernation may be disrupted where anthropogenic food is regularly available (Gazzard & Baker, 2020), and the nutritional quality of such food may also be inadequate (Gimmel, Eulenberger & Liesegang, 2021). In addition, artificial food often attracts several individuals to the same location, which may increase intra-specific aggression; although this is known to happen at feeding stations, it is not known whether this also extends to artificial refuges. Nonetheless, proximity to a food source may be desirable in those instances where hedgehogs may be reluctant to move far from a nest site, for example, during breeding when vulnerable young are present, and during hibernation when natural food availability is low. Similarly, staying close to a nest site would potentially be important where nest boxes themselves are a limiting resource. Whether this is an issue is, however, equivocal: for example, the householders in this study collectively reported an average of 0.6 other nest sites within their gardens, in locations that are likely to be present in a broad range of other gardens (*e.g.*, in vegetation and compost heaps, as well as underneath woodpiles, sheds and decking). Furthermore, the quantity of urban habitat within 500 m of the garden, and the distance to the nearest area of arable land, woodland or grassland, had no effect on nest box use suggesting that these other habitats are not critically important as potential nesting locations.

One of the major factors thought to affect urban hedgehog populations is patterns of connectivity between neighbouring back gardens but also from an individual householder’s front garden to their back garden (Gazzard et al., 2021; App et al., 2022; Gazzard, Yarnell & Baker, 2022). In this study, nest box use was not significantly affected by the number of neighbouring back gardens which were accessible to hedgehogs from the respondent’s own garden, but front-to-back access was significantly positively correlated with all four patterns of nest box use. Both results could potentially be explained by the fact that most householders put out boxes once they knew hedgehogs were already visiting; consequently, access into the respondent’s garden was already possible. Alternatively, front-to-back access could simply be a proxy for houses with larger gardens: in the UK, detached and semi-detached houses are typically associated with larger gardens which permit access down both or one the sides of the house, respectively; terraced houses typically have the smallest sized gardens, and access from the front to the back is not always possible. However, it has been noted that front-to-back access also significantly decreases the proportion of time hedgehogs spend in back gardens (Gazzard, Yarnell & Baker, 2022). Although the underlying reason for this is not known, it could suggest that front gardens contain important resources not present in back gardens, or that this facilitates movement through the landscape (particularly between blocks of houses separated by roads) and perhaps even helps animals avoid one another when foraging. More detailed information is therefore required on how key resources are distributed throughout the urban landscape, but also the patterns of behaviour exhibited by hedgehogs in different types of gardens.

Given the potential vulnerability of animals that are sleeping, hibernating or which have dependent young, hedgehogs would be expected to select locations which reduce the risk of detection by predators (Evans et al., 2002), accidental disturbance and which also offer protection from inclement weather conditions; refuges located in sheltered locations are likely to experience reduced exposure to rain, wind and direct sunshine, and may more closely mimic natural nesting locations (Morris, 2018). Accordingly, nest boxes in the current study were more likely to have been used by hedgehogs when they were positioned in sheltered locations, such as under shrub cover, and less likely to have been used when entrances faced into the open (*i.e.,* facing towards the middle of the garden); these relationships were generally consistent for all nesting types with the exception of sheltered locations during hibernation and the orientation of the box’s entrance during breeding. It may be the case that during breeding and hibernation, the influence of other factors are of greater relative importance, aligning with the specific needs and/or vulnerabilities of hedgehogs at these times.

During the hibernation period, there is a risk that exceptionally low temperatures within nests could trigger thermogenesis (Malan, 2010) leading to the utilisation of brown fat stores (Nedergaard & Cannon, 1990) which are critical for rapid metabolism during arousals from torpor (Morris, 2018). Indeed, in hedgehogs, hibernacula temperatures <0 °C have been associated with increased oxygen consumption and shorter periods of torpor, compared to individuals in nests maintained at temperatures >0 °C (Soivio, Tähti & Kristoffersson, 1968). In this study, nest boxes located on hardstanding, within close proximity (<5 m) to buildings and those with entrances oriented to the south were more likely to have been used for hibernation (although summer day nesting was also positively influenced by the former variables). Temperatures of hardstanding and building surfaces tend to be higher than those measured on soil, grass or other green areas due to their greater ability to absorb and retain solar radiation (see Bowler et al., 2010; Loughner et al., 2012). Additionally, when entrances are oriented east or south, internal nest temperatures can be warmer than in nest boxes facing other orientations (Ardia, Pérez & Clotfelter, 2006; Butler, Whitman & Dufty Jr, 2009). As such, it is possible that the thermal properties of surrounding substrates and ambient sunshine could positively influence temperature profiles within nest boxes during the hibernation period, although this requires verification as well as investigation of how thermal profiles may be affected by the design of the next box itself.

Hedgehogs were also significantly less likely to have used a nest box for hibernating on sites where the respondent owned a dog. Hibernating animals are presumably less responsive to predation attempts or disturbances (Boyles et al., 2020) since it takes typically >5 h for hedgehogs to fully arouse from torpor (Morris, 2018). They may therefore seek to hibernate in locations where predators are less likely to occur. The presence of badgers and/or foxes did not, however, have a marked effect on nest box use during hibernation, although it must be noted that badgers do not often leave their setts over winter (Fowler & Racey, 1988), and we were not able to investigate the effect of foxes alone as only a low number of respondents reported having sighted them in their garden. However, it is reasonable to assume that hedgehog boxes are often likely to be sited in gardens where foxes are present, given their agility and widespread distribution in urban areas in the UK (Scott et al., 2014). Similarly, both foxes and badgers are likely to be attracted to gardens where food is put out for hedgehogs by the householder. At the present time, however, there are few data available on the frequency with which hedgehogs, foxes and badgers interact with one another in residential gardens, the manner of these interactions nor the effectiveness of anti-predation features of hedgehog nest boxes such as integral bases, external tunnels or internal partitions (see Bailey & Bonter, 2017).

In this study, we were not able to investigate in detail how the design of nest boxes influenced their use by hedgehogs since many design-related variables had to be excluded due to issues arising from multicollinearity. Of the variables that were included in the analyses, the external dimensions, internal volume and primary construction materials of boxes were not important factors affecting nest box use, whereas the type of nest box and whether it was raised off the ground had significant impacts. First, homemade nest boxes were more likely to have been used than commercially available nest boxes for winter day nesting and hibernation. This could possibly be associated with parameters that were not measured in this study, such as the age, condition, thickness or colour of the materials used to construct homemade boxes. For example, it has been demonstrated that dark-green wooden nest boxes experience greater average daily temperatures when compared to boxes painted with lighter colours (Griffiths et al., 2017); it could be possible that homemade nest boxes were more likely to have been painted in such a way that influenced nest box selection during colder periods. Second, boxes that were raised off the ground were significantly more likely to have been used for summer day nesting and hibernation. However, it is not clear whether the latter was representative of a specific design feature (*i.e.,* legs attached to the base), placement decision (*e.g.*, nest box was elevated on bricks), or even a proxy of such boxes possessing bases (by default, a box raised off the ground would have a solid base). Further investigation is needed to determine any preferences for, and the effects of, various nest box design features.

Resources provided by householders strongly influenced nest box use, with the provisioning of bedding and food associated with the largest odds ratios across all models. The provision of bedding materials (*e.g.*, leaves, hay and/or shredded newspaper) within nest boxes significantly positively influenced their use for day and hibernation nesting, but had a (nonsignificant) negative effect in the context of breeding. During reproduction, females with dependent young are particularly sensitive, and mothers may abandon breeding nests and/or kill their young if their nests are disturbed (Morris, 2018). Typically, such disturbance is associated with human activities (*e.g.*, garden maintenance, dog walkers) but could potentially arise because of intra-specific interactions, although there is no definitive evidence of this. Consequently, it may be that pregnant females consider the presence of bedding material to be an indication of the presence of other hedgehogs, particularly if the box retains the scent of individuals who have visited previously.

In studies of naturally constructed nests, the leaves of broadleaved trees appeared to be preferred, especially as these can be “woven” to create a layered structure which is thought to help maintain the temperature within the nest whilst allowing gaseous transfer (Morris, 2018). Unfortunately, broadleaved trees can often be removed from urban areas in the UK because of, *e.g.*, human safety concerns and the risk posed to buildings (Pauleit, Ennos & Golding, 2005; Andrew & Slater, 2015). In addition, fallen leaves in gardens are often removed by householders for aesthetic reasons. This might consequently be linked to the positive association between nest box use (for summer and winter day nesting, and hibernation) and the active provision of bedding material by humans. However, different nesting materials are likely to vary with respect to their thermal properties (Corrales-Moya et al., 2021), longevity (Hebda, Kandziora & Mitrus, 2017) and/or influence on ectoparasite presence (Reynolds et al., 2019). In addition, some man-made materials may contain toxic compounds that could affect survival (Mukai et al., 2014). Additional research is therefore required to examine which materials are being used by hedgehogs within nest boxes, how this relates to material availability in the wider environment and how nest structure ultimately affects their behaviour and success. Such research would help to identify the most suitable materials which householders should provide, if this was deemed necessary (*sensu* Slobodník et al., 2017), but also help to determine suitable box cleaning regimes (*e.g.*, Tomás et al., 2007).

## Conclusions

The use of an online questionnaire survey of householders enabled the rapid collection of a large quantity of information relating to factors affecting nest box use by hedgehogs, but was associated with limitations. First, we were only able to investigate factors associated with whether a box had ever been used, rather than their frequency of use. Second, several variables that were significantly associated with the increased use of boxes could have represented a form of reporting bias whereby respondents who were especially ‘hedgehog-friendly’ may have been more likely to monitor their boxes for hedgehog activity. Whilst acknowledging the limitations of these data, the results indicate moderate to high uptake rates of hedgehog boxes for nesting. Subtle differences in the factors associated with the four patterns of nesting were identified relating to nest box placement, resource provisioning and site-based features; in general terms, householders might be able to improve the likelihood that their nest boxes are used by hedgehogs by placing them under shelter, ensuring that their gardens are sufficiently accessible from the front garden to the back, and providing additional resources such as food and bedding. In some seasons, including over winter, positioning the nest box on hardstanding and/or closer (<5 m) to a building, and ensuring that the box entrance does not face the open, may increase the chances of it being used. The drivers behind such placement ‘preferences’ are, however, unclear, and further research is needed to investigate these factors in more detail.

### Future research recommendations

Although questionnaire surveys represent a mechanism for rapidly collecting large volumes of data, they are susceptible to reporting biases that may exaggerate the importance of hedgehog boxes as nesting sites. Future studies, therefore, need to adopt experimental or quasi-experimental approaches whereby householders are recruited in a more randomised manner. Such studies need to ensure that all householders monitor their boxes continuously so that definitive data on patterns of use can be obtained; this could involve existing technologies, such as commercially available motion-activated cameras, or the development of new approaches such as cameras or other devices mounted inside nest boxes. Furthermore, additional monitoring techniques and/or novel experimental approaches are required to quantify how the internal conditions of boxes are affected by their design and placement, and the relative vulnerability of boxes to other species; choice experiments within gardens would help to identify whether hedgehogs select particular box designs or types of bedding. Finally, the pattern of use of nest boxes must be considered in the context of natural nest site availability, *i.e.,* are nest boxes used more frequently where natural nest sites are limited? Consequently, field studies are required to quantify the frequency of use of artificial refuges relative to other sites, but which also compare the success of hedgehogs in nest boxes *versus* other nesting sites in the context of, for example, over-winter survival rates and reproductive success; ideally, such studies should consider both urban and rural landscapes.

##  Supplemental Information

10.7717/peerj.13662/supp-1 Supplemental Information 1 Questionnaire

10.7717/peerj.13662/supp-2 Supplemental Information 2 Nest box questionnaire data

## Additional Information and Declarations

### Data Availability

The following information was supplied regarding data availability: A text copy of the questionnaire and anonymised questionnaire results are available in the Supplementary Files.
