# Supplementary material for: What makes a house a home? Nest box use by West European hedgehogs (Erinaceus europaeus) is influenced by nest box placement, resource provisioning and site-based factors
Source: PeerJ. 2022 Jul 4;10:e13662. doi: 10.7717/peerj.13662 (PMC9261924; doi:10.7717/peerj.13662)
Supplement: Supplemental Information 1 [file peerj-10-13662-s001.docx]

This is the first ever national census of hedgehog houses, brought to you by Hedgehog Street: a national campaign from People's Trust for Endangered Species and the British Hedgehog Preservation Society. This survey is in partnership with the University of Reading and Warwickshire Wildlife Trust.

There is a lot of advice about how best to use hedgehog houses, but we need to do some research to be sure that this advice is well-founded and appropriate. The aim of this survey is to collect evidence that will help to guide people on how best to use hedgehog houses to help hedgehogs, based on the experiences of the thousands of you that already have them. However, please do not infer any guidance or advice on using hedgehog houses from the wording of the questions in the survey; we are just asking about your past experiences, not suggesting any particular course of action. So please do not disturb any hedgehogs or nests in order to complete this survey. We will share the results with you and give guidance once the data have been collected and analysed.

If you would like to provide information about more than one hedgehog house in your garden, and if you have the time fill, please in the survey for each house separately. If this is too much not to worry, please just select those that you know have been definitely used by hedgehogs. But, just make sure that in the final question “Any other comments” please write “2nd house” or “4th house” etc.

Thank you very much!

**PART 1:**

Q1. First name [short text answer]

Q2. Surname [short text answer]

Q3a. Address [short text answer] (was not mandatory)

Q3b. Postcode [short text answer]

Q4. Email [short text answer]

Q5. Are you registered as a Hedgehog Champion on Hedgehog Street? [Yes / No]

*All of the questions in this survey relate to hedgehog houses designed for animals to nest in, not feeding stations designed to shelter hedgehogs whilst they eat supplementary food. We are interested only in the former, which should not provide food.*

**PART 2:**

Q6. Is your hedgehog house commercially available or is it homemade?

[If person responds commercially available they need to be directed to **Question 7**]

[If person responds homemade they need to be directed to **Question 8**]

**Commercially available hedgehog houses**

Q7. If your house is commercially available, which design is it? (images were provided)

1. Chapelwood [Tick response]
2. Coopers of Stortford [Tick response]
3. Eco-plate Royal [Tick response]
4. Gardman Norfolk [Tick response]
5. Hogilo [Tick response]
6. Hogitat [Tick response]
7. Home with inbuilt cameras [Tick response]
8. Igloo domed [Tick response]
9. Orkney [Tick response]
10. RSPB [Tick response]
11. Schwegler [Tick response]
12. Tom Chambers [Tick response]
13. UK garden supplies [Tick response]
14. Waitrose wicker [Tick response]
15. Wooden domed [Tick response]
16. Wudwerx [Tick response]
17. Other (if other, please specify and add a link to the supplier)

**DIY hedgehog houses**

Q8. What is the main material the box is made from? Please tick one option.

1. Timber [Tick response]
2. Plywood / plyboard [Tick response]
3. Plastic [Tick response]
4. Concrete [Tick response]
5. Brick [Tick response]
6. Other [Tick response]

Q9. Please provide the approximate dimensions of the box, in centimetres.

1. Width (side to side) [Number]
2. Height (top to bottom) [Number]
3. Depth (front to back) [Number]

Q10. Does your box have any of the following features?

1. A base or floor [Tick response]
2. An external tunnel entrance [Tick response]
3. An internal tunnel or partition [Tick response]
4. An air vent [Tick response]
5. A waterproof lining [Tick response]

[Now all to be directed to **Question 11**]

**PART 3:**

Q11. Is your hedgehog house in the front or back garden?

1. Front garden [Tick response]
2. Back garden [Tick response]

Q12. Approximately when did you install your hedgehog house? [Month and Year]

Q13. Did you put your hedgehog house in your garden:

1. BEFORE you knew whether hedgehogs might be visiting? [Tick response]
2. AFTER you knew that hedgehogs were visiting? [Tick response]

*Questions 14-17 relate to whether hedgehogs have used your hedgehog house for resting, breeding and / or hibernating. For each question, we would also like to know what evidence you may have for substantiating your answers. However, please be aware that this is a survey of historic actions only.* ***We request that you avoid checking or disturbing hedgehogs currently residing in your hedgehog house(s) as this may affect their behaviour and lead to abandonment of the nest box, abandonment of hoglets or unnecessary disturbance during hibernation.***

Q14a. Since it was installed, how many years do you think that the hedgehog house has been used for **RESTING DURING THE DAYTIME** between the months of **March and October** (please answer “don’t know” if applicable): [short text answer]

Q14b. What evidence do you have for this (please tick all that apply)?

1. I saw a hedgehog using the entrance [Tick response]
2. I saw one inside [Tick response]
3. I recorded activity on a wildlife camera [Tick response]
4. I placed an object in front of the entrance and it was displaced [Tick response]
5. I have other evidence: please describe [short text answer]
6. Not applicable [Tick response]

Q15a. Since it was installed, how many years do you think that the hedgehog house has been used for **RESTING DURING THE DAYTIME** between the months of **November and February** (please answer “don’t know” if applicable): [short text answer]

Q15b. What evidence do you have for this (please tick all that apply)?

1. I saw a hedgehog using the entrance [Tick response]
2. I saw one inside [Tick response]
3. I recorded activity on a wildlife camera [Tick response]
4. I placed an object in front of the entrance and it was displaced [Tick response]
5. I have other evidence: please describe [short text answer]
6. Not applicable [Tick response]

Q16a. Since it was installed, how many years do you think that the hedgehog house has been used for **BREEDING** (please answer “don’t know” if applicable): [short text answer]

Q16b. What evidence do you have for this (please tick all that apply)?

1. I saw a mother and babies using the entrance [Tick response]
2. I saw a mother and babies inside [Tick response]
3. I recorded a mother and babies on a wildlife camera [Tick response]
4. I have other evidence: please describe [short text answer]
5. Not applicable [Tick response]

Q17a. Since it was installed, how many years do you think that the hedgehog house has been used for **HIBERNATING** (please answer “don’t know” if applicable): [short text answer]

Q17b. What evidence do you have for this (please tick all that apply)?

1. I saw a hibernating hedgehog inside [Tick response]
2. I have other evidence: please describe [short text answer]
3. Not applicable [Tick response]

Q18. In the past, have you ever provided any of the following inside your hedgehog house (please select all that apply)?

1. Food [Tick response]
2. Water [Tick response]
3. Artificial bedding (e.g. newspaper) [Tick response]
4. Natural bedding (e.g. leaves, hay) [Tick response]
5. Other [Tick response]

*The following questions relate to bedding material you may have found in your hedgehogs box in two time periods: March-October and November-February. In each case, we would like you to list the range of natural (e.g. leaves, grass) and man-made materials (e.g. newspaper, rubbish) that the animals may have used to construct their nests. When describing the materials you have found, please be as accurate as possible. For example, it would help if you were able to identify the species of leaves used if possible.*

Q19a. Have you ever found bedding material in your hedgehog box that the animals would have used between the months of **March and October?**

1. No [Tick response]
2. Yes – please list the range of materials that the hedgehogs used to construct the nest [short text answer]

Q19b. Have you ever found bedding material in your hedgehog box that the animals would have used between the months of **November and February?**

1. No [Tick response]
2. Yes – please list the range of materials that the hedgehogs used to construct the nest [short text answer]

Q20. Where is your hedgehog house positioned? Please select all that apply:

1. On a natural substrate [Tick response]
2. On hardstanding [Tick response]
3. In a sheltered spot (e.g. under vegetation) [Tick response]
4. In the open [Tick response]
5. Near my house (<5m away) [Tick response]
6. Away from my property (>5m away) [Tick response]
7. It is raised off the floor (e.g. it has its own legs) [Tick response]

Q21. Does the entrance of the hedgehog house:

1. Face a wall or fence [Tick response]
2. Lie parallel to a wall or fence [Tick response]
3. Face into a bush [Tick response]
4. Face into the open [Tick response]
5. Other (please specify): [short text answer]

Q22. Does the entrance of the hedgehog house face:

1. North [Tick response]
2. South [Tick response]
3. East [Tick response]
4. West [Tick response]

Q23. Since your hedgehog house has been installed, are you aware of hedgehogs (a) resting during the daytime, (b) breeding or (c) hibernating in **ANY OTHER LOCATION** in your garden (please put at least one answer in each column)?

|  | (a) Resting  during daytime | (b) Breeding | (c) Hibernating | (d) Not applicable |
| --- | --- | --- | --- | --- |
| Yes – under the shed |  |  |  |  |
| Yes – in compost heap |  |  |  |  |
| Yes – in woodpile |  |  |  |  |
| Yes – in bushes |  |  |  |  |
| Yes – under decking |  |  |  |  |
| Yes – inside building |  |  |  |  |
| Yes - other |  |  |  |  |
| No |  |  |  |  |

Q24. Do you think that your hedgehog house is the only reasonable site in your garden where hedgehogs could rest / breed / hibernate? [Yes / No]

Q25a. How many front gardens does your front garden border? [Number]

Q25b. Of these, how many do you think hedgehogs could access from your front garden? [Number]

Q26. Can hedgehogs access your back garden from your front garden? [Yes / No]

Q27a. How many back gardens does your back garden border? [Number]

Q27b. Of these, how many do you think hedgehogs could access from your back garden? [Number]

Q28. How often do you think these mammal species visit your garden (please tick one option for each of the four species indicated)?

|  | Daily | Weekly | Monthly | Every 3  months | Every 6  months | Less  regularly | Never |
| --- | --- | --- | --- | --- | --- | --- | --- |
| Badger |  |  |  |  |  |  |  |
| Fox |  |  |  |  |  |  |  |
| Hedgehog |  |  |  |  |  |  |  |
| Rat |  |  |  |  |  |  |  |

Q29. Please indicate the approximate percentage area of your BACK GARDEN covered by each of the following features (adding up to 100%). If your back garden does not have any of the features listed, please enter a zero in the appropriate cell.

| **Feature** | **% coverage** |
| --- | --- |
| Lawn |  |
| Paving/gravel |  |
| Flowerbed(s) |  |
| Shrubs |  |
| Decking |  |
| Shed with cavity beneath |  |
| Compost heap |  |
| Woodpile |  |
| Wild area |  |
| Vegetable patch |  |
| Pond |  |
| Other (please describe) |  |

Q30. Do you ever put out any of the following foods? Please tick all that apply.

1. Food for hedgehogs in a covered feeding station [Yes / No]
2. Food for hedgehogs not in a covered feeding station [Yes / No]
3. Food for other mammals such as foxes or badgers [Yes / No]
4. Food for birds on the ground [Yes / No]

Q31. If you do put out food for hedgehogs, how far away (metres) **from the hedgehog house** is the site where you put out the food? [Number]

Q32. How many pet dogs do you own which have access to your garden? [Number]

Q33. Space for additional comments.
